# Label-free imaging of lipid-rich biological tissues by mid-infrared photoacoustic microscopy

**DOI:** 10.1117/1.JBO.25.10.106506

**Published:** 2020-10-28

**Authors:** Yun He, Junhui Shi, Miguel A. Pleitez, Konstantin Maslov, Daniel A. Wagenaar, Lihong V. Wang

**Affiliations:** a Washington University in St. Louis, Department of Biomedical Engineering, St. Louis, Missouri, United States; b California Institute of Technology, Andrew and Peggy Cherng Department of Medical Engineering, Department of Electrical Engineering, Caltech Optical Imaging Laboratory, Pasadena, California, United States; c California Institute of Technology, Division of Biology and Biological Engineering, Pasadena, California, United States

**Keywords:** photoacoustics, microscopy, mid-infrared, lipids

## Abstract

**Significance:** Mid-infrared (IR) imaging based on the vibrational transition of biomolecules provides good chemical-specific contrast in label-free imaging of biology tissues, making it a popular tool in both biomedical studies and clinical applications. However, the current technology typically requires thin and dried or extremely flat samples, whose complicated processing limits this technology’s broader translation.

**Aim:** To address this issue, we report mid-IR photoacoustic microscopy (PAM), which can readily work with fresh and thick tissue samples, even when they have rough surfaces.

**Approach:** We developed a transmission-mode mid-IR PAM system employing an optical parametric oscillation laser operating in the wavelength range from 2.5 to 12  μm. Due to its high sensitivity to optical absorption and the low ultrasonic attenuation of tissue, our PAM achieved greater probing depth than Fourier transform IR spectroscopy, thus enabling imaging fresh and thick tissue samples with rough surfaces.

**Results:** In our spectroscopy study, the ${\mathrm{CH}}_{2}$ symmetric stretching at $2850\;{\mathrm{cm}}^{-1}$ (3508 nm) was found to be an excellent source of endogenous contrast for lipids. At this wavenumber, we demonstrated label-free imaging of the lipid composition in fresh, manually cut, and unprocessed tissue sections of up to 3-mm thickness.

**Conclusions:** Our technology requires no time-consuming sample preparation procedure and has great potential in both fast clinical histological analysis and fundamental biological studies.

## 1 Introduction

Imaging technologies exploiting the vibrational transition of biomolecules,^1^^,^^2^ such as Fourier transform infrared (FTIR) spectroscopy^3^ and stimulated Raman scattering (SRS) imaging,^4^ provide rich and specific information about tissue’s biochemical composition in a label-free manner.^5^^–^^10^ Because of their ability to classify biomolecules (e.g., glycogen, proteins, lipids, or nucleic acids),^11^^,^^12^ these technologies have many biomedical applications, such as analyzing clinical biopsy samples *ex vivo*^13^^–^^16^ and studying disease progression with tissue sections taken from animal models.^17^^,^^18^ However, their broader translation is still inhibited by certain intrinsic limitations. Traditional FTIR is mainly limited to thin and dried samples^16^^,^^18^^–^^21^ because the light it detects is heavily attenuated by biological tissue, which is highly absorbing in the mid-IR range.^9^ An emerging FTIR technique, attenuated total reflection (ATR), can image thick samples and live cells in an aqueous environment.^10^^,^^22^^–^^25^ However, it still requires time-consuming sample preparation and an expensive slicing instrument to produce an extremely flat surface,^26^^–^^28^ because its maximum probing depth is only ∼3  μm.^22^^,^^25^^,^^29^ SRS imaging is capable of imaging unprocessed tissues, but it requires a complicated detection device and suffers from poor sensitivity and possible photodamage^30^ since it relies on the weak Raman scattering effect.^2^

Here, we introduce mid-IR photoacoustic microscopy (PAM) as a vibrational imaging technique for fresh and thick tissue samples with rough surfaces. In PAM, the absorption of a short-pulsed laser generates ultrasonic waves via transient thermoelastic expansion.^31^ After propagating through tissue, these ultrasonic waves are detected by the transducers as PA signals, whose amplitude is proportional to the absorbed optical energy (positive contrast).^32^ Since biological tissues usually absorb strongly in the mid-IR range^3^ and attenuate ultrasound much less than light,^33^ mid-IR PAM offers a better signal-to-noise ratio than FTIR, which relies on detecting the photons remained after heavy attenuation in tissue (negative contrast). The combination of ultrasonic detection and positive-signal sensing enables mid-IR PAM to probe more deeply in tissue, making it more suitable for thick samples with rough surfaces and cell cultures in aqueous media. In this paper, we demonstrate mid-IR PAM by imaging manually cut tissue samples without additional preparation.

## 2 Results

**Fig. 1:**
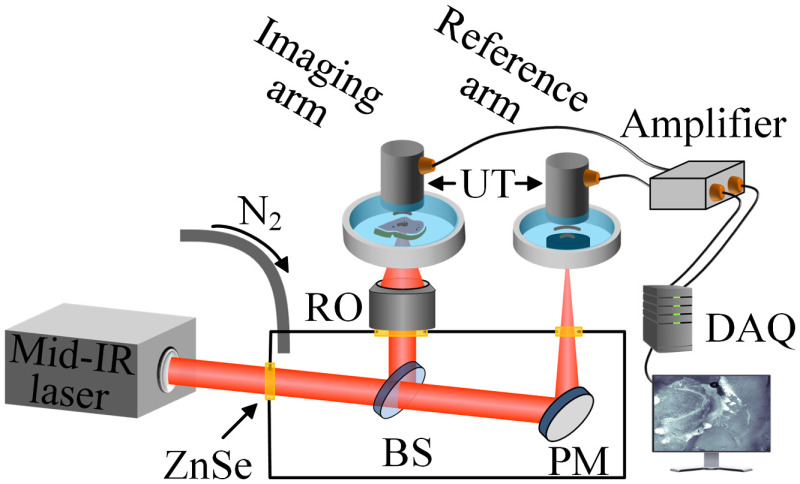
Schematic of the mid-IR PAM system. PA signals from the tissue sample and the carbon black are acquired concurrently to produce a calibrated reading of the tissue’s absorption property. BS, beam splitter; DAQ, data acquisition unit; PM, parabolic mirror; RO, reflective objective; UT, ultrasonic transducer.

Our mid-IR PAM employed an optical parametric oscillation (OPO) laser (NT270, EKSPLA) as the light source (Fig. 1). It provided ∼10-ns laser pulses at the wavelength tunable from 2.5 to 12  μm. The output laser beam was split into two parts by a germanium (Ge) beam splitter, with one part going to the imaging arm and the other to the reference arm. In the imaging arm, the laser beam was focused onto the tissue sample by a reflective objective with a numerical aperture of 0.52 (50102-02, Newport). A pulse energy of 0.5  μJ was typically used for tissue imaging. The excited PA waves were detected by a focused ultrasonic transducer (V324-SM, Olympus: 25 MHz central frequency, aperture 6.35 mm, focal length 12.7 mm) placed on top of the tissue sample. Deionized water was used for acoustic coupling, and the tissue sample was attached to a zinc selenide (ZnSe) substrate (largely transparent in the mid-IR range) embedded at the bottom of the water tank. The detected PA signal was first amplified by two radio-frequency amplifiers in series (ZFL-500LN+, Mini-circuits), and then acquired by a data acquisition unit (Razor 14, Gage) at a 200-MHz sampling rate.

Because the OPO laser’s output energy is highly dependent on its wavelength, the reference arm was used to correct for this variation. The other part of the laser beam transmitted through the Ge beam splitter was focused, by an off-axis parabolic mirror (MPD127254-90-M01, Thorlabs), onto a strong absorber, a slab of carbon black, whose absorption spectrum in the mid-IR is relatively flat and well-studied.^34^^,^^35^ Then the PA signal from the carbon black was similarly amplified and digitized such as that from the imaging arm, and it was used as a reference reading of the OPO laser’s output energy when normalizing the sample’s PA signal. To reduce the laser’s absorption by atmospheric water vapor and carbon dioxide, most of the laser beam path was enclosed in a nitrogen-infused box, with ZnSe windows embedded on its walls as the laser input and output ports. The whole system was automated by a microcontroller board (Arduino Mega 2560, Arduino) and operated through an interface programed in LabVIEW.

**Fig. 2:**
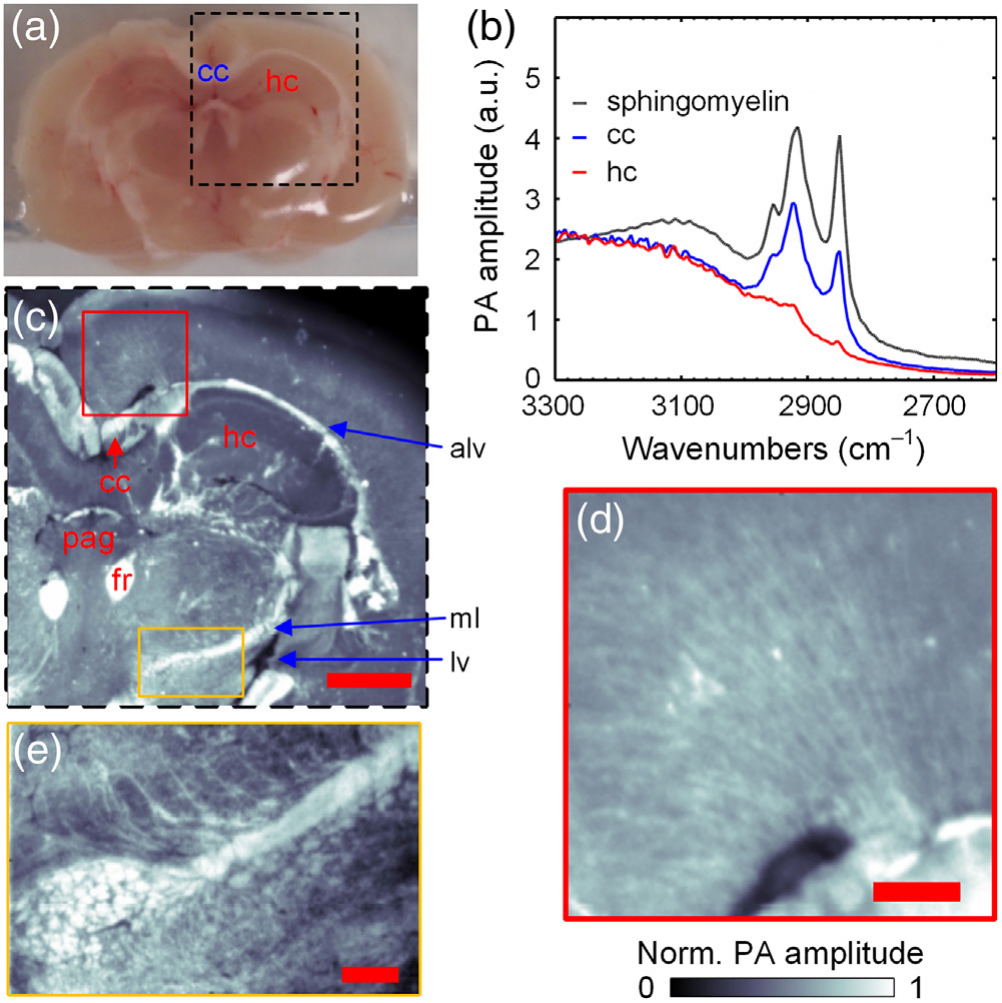
Mid-IR PAM imaging of a mouse brain slice. Mid-IR PAM imaging of a mouse brain slice. (a) A photo of the thick manually cut brain slice. (b) PA spectra measured at the two sites on the brain slice indicated by the acronyms in (a). The PA spectrum of the sphingomyelin solution (black) is also plotted here. cc, corpus callosum (blue) and hc, hippocampus (red). (c) PAM image of the region of interest denoted by the dashed box in (a). alv, alveus; fr, fasciculus retroflexus; lv, lateral ventricle; ml, medial lemniscus; and pag, periaqueductal gray nucleus. Scale bar, 1 mm. (d) Enlarged view of the retrosplenial area [red box in (c)]. (e) Enlarged view of the superior cerebellar peduncle [yellow box in (c)]. Scale bars in (d) and (e), 200  μm.

We demonstrated PAM’s capability by imaging thick and fresh tissue samples with rough surfaces. First, we applied our mid-IR PAM to image a fresh coronal section of a mouse brain, an organ rich in lipids, such as the myelin sheath surrounding the axons. A photo of this ∼2-mm-thick brain slice is shown in Fig. 2(a). All animal experiments were carried out in compliance with the laboratory animal protocols approved by the Institutional Animal Care and Use Committee of California Institute of Technology. First, fresh organs were procured from female mice (ND4 Swiss webster, Envigo; 8 to 12 weeks) after being euthanized by a carbon dioxide overdose. Then, all tissue sections were acquired by simply slicing the harvested organ manually with a pair of blades sandwiching a spacer of a certain thickness. No additional processing was performed afterward, and the sliced tissue samples were attached to the ZnSe substrate and immersed in deionized water for imaging. Figure 2(b) shows the PA spectrum acquired from two regions of interest on this brain slice, as labeled in Fig. 2(a). The data from the corpus callosum, which are composed of myelinated fiber bundles, show peak absorption at 2930 and $2850\;{\mathrm{cm}}^{-1}$ (3413 and 3508 nm), which both are signature lipid absorption bands corresponding to asymmetric and symmetric stretching of the ${\mathrm{CH}}_{2}$ group, respectively. In comparison, the spectrum from the hippocampus, which mainly consists of gray matter, demonstrates much weaker absorption at these two wavenumbers. Also shown is the PA spectrum of sphingomyelin (567706-100MG, Sigma-Aldrich), the main component of the myelin sheath, in a methanol solution, and its peaks match well with these of the corpus callosum. In addition, we compared our measured spectrum of sphingomyelin with that acquired by a commercial ATR-FTIR spectrometer (Thermo Nicolet Nexus 470 spectrometer, ThermoFisher) (Fig. 5). Our data’s good agreement with the ground truth confirms our mid-IR PAM’s ability to capture the biomolecule’s absorption property in this range. We found that the ${\mathrm{CH}}_{2}$ symmetric stretching at $2850\;{\mathrm{cm}}^{-1}$ offers a better contrast for lipids in biological tissues, despite the slightly stronger absorption at $2920\;{\mathrm{cm}}^{-1}$.

With this finding, we acquired a mapping of the lipid composition in this brain slice at $2850\;{\mathrm{cm}}^{-1}$ by point-by-point raster scanning with a 2.5-μm step size. No data averaging were necessary. The system’s theoretical lateral resolution was calculated to be 3.37  μm at this wavenumber. As shown in Fig. 2(c), regions rich in myelinated fiber bundles, such as the alveus, fasciculus retroflexus, and medial lemniscus, appear very bright and well defined. Some other regions that mainly consist of neuron somas and glial cells, such as the periaqueductal gray nucleus, appear darker. Nevertheless, their PA signal amplitude is still significantly greater than the cavity structure, the lateral ventricle, which looks like a void with clear boundaries. The contrast-to-noise ratio for the cortex region is ∼35. In the close-up image of the retrosplenial area [Fig. 2(d)], we can clearly see the densely packed myelinated nerve fibers projected from the cortex. In comparison, the close-up image of the superior cerebellar peduncle shows thicker nerve fiber bundles [Fig. 2(e)].

**Fig. 3:**
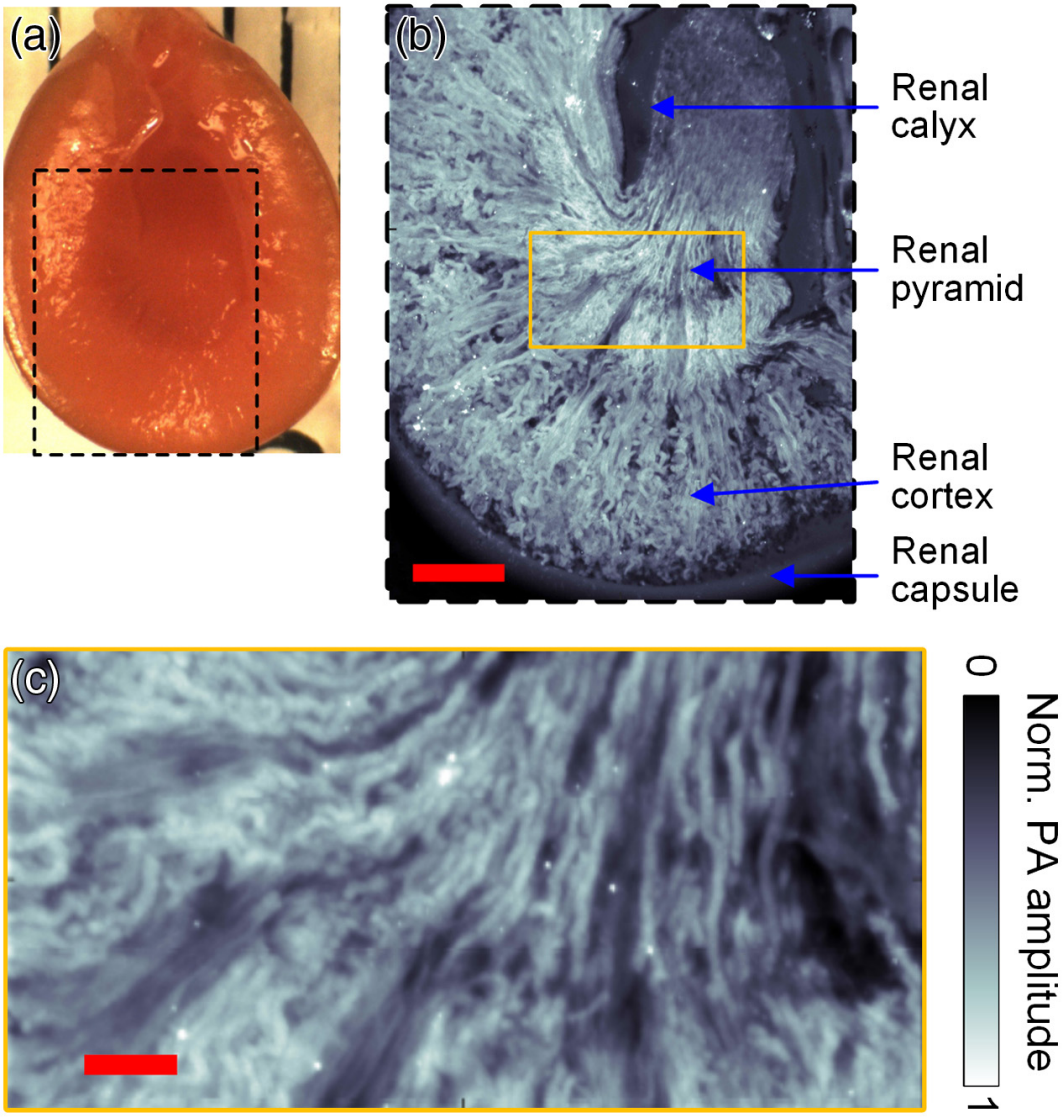
Mid-IR PAM imaging of a mouse kidney slice. (a) A photo of the thick manually cut kidney sample. (b) Image of the region of interest denoted by the dashed box in (a), showing all the major structures of the kidney as annotated in the panel above. Scale bar, 1 mm. (c) Enlarged view of part of the renal pyramid as illustrated in the yellow box in (b). Scale bar, 200  μm.

We also imaged a 2- to 3-mm-thick fresh section of a mouse kidney [Fig. 3(a)] at $2850\;{\mathrm{cm}}^{-1}$. As shown in Fig. 3(b), the kidney anatomy can be clearly resolved by mapping the lipid composition. The renal pyramid and cortex, both rich in urine-collecting tubules, appear very bright in this image, whereas the renal calyx chamber is much darker and well defined from the renal pyramid projecting into it. There is also a clear boundary between the parenchyma and the renal capsule encapsulating it. In the close-up of the renal pyramid [Fig. 3(c)], we can also resolve the radially diverging tubules within.

**Fig. 4:**
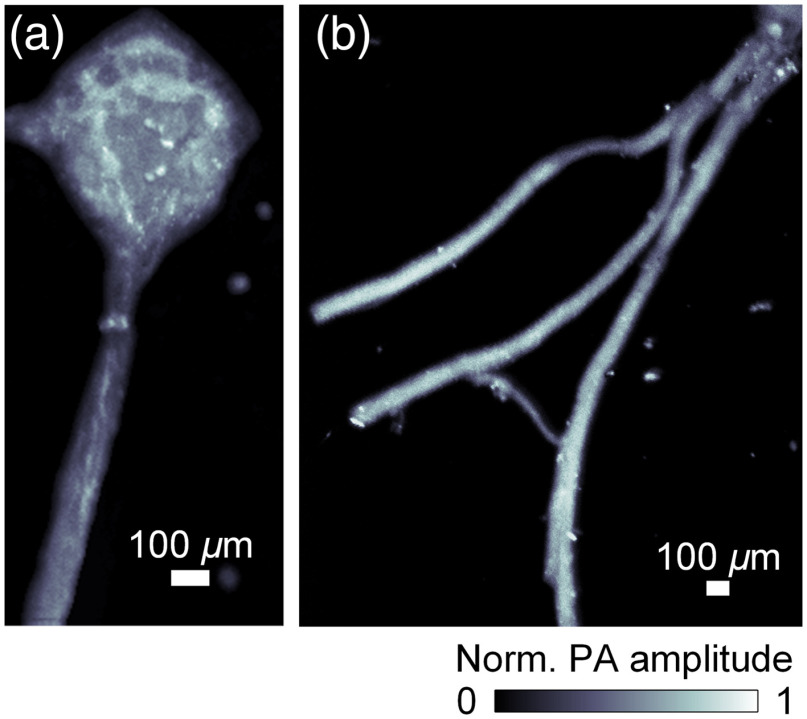
Mid-IR PAM imaging of nerve samples covered by muscle tissues. (a) Image of the body ganglion and the nerve cord. (b) Image of the peripheral nerves projecting out of the ganglion node.

Finally, we explored our system’s ability to image peripheral nerves A segmental ganglion from a medicinal leech (Hirudo verbana) with its ventral nerve cord [Fig. 4(a)] and another ganglion node with its two lateral nerves [Fig. 4(b)] were dissected and imaged while covered by a 3-mm-thick layer of leech dorsoventral muscle tissue. The fine structures of the ganglion node are shown in Fig. 4(a). Figure 4(b) also demonstrates sufficient contrast for imaging the peripheral nerves over the background of muscular tissue.

## 3 Discussion

Although mid-IR imaging is a promising imaging technology for extracting the rich biochemical information of tissue, its critical limitations must be surmounted to enable its broader clinical and research use. In this paper, we present mid-IR PAM, which has successfully imaged fresh and thick biological samples that were manually sliced from tissue without additional processing, significantly reducing the sample-preparation burden. Our mid-IR PAM works on a positive-contrast basis, which is very suitable for working with highly absorbing biological tissues. Exploiting the ${\mathrm{CH}}_{2}$ stretching transition, we acquired detailed mapping of the lipid composition in tissue sections. This technology can be applied in a wide range of clinical examinations and preclinical studies, such as monitoring the abnormal lipid component in the Alzheimer’s disease model.

A natural next step of our study is to extend the system to other molecules’ signature bands in the tuning range of our OPO laser, such as 1500 to $1700\;{\mathrm{cm}}^{-1}$ for protein and $1084\;{\mathrm{cm}}^{-1}$ for nucleic acids. Our mid-IR PAM obviates the need for time-consuming sample processing, but the image acquisition time for a field of view like Fig. 2(c) ($∼5\times 5\;{\mathrm{mm}}^{2}$ field of view) is ∼1  h, which is limited by the pulse repetition rate of our OPO laser (1 kHz). Future work can implement fast imaging techniques such as multifocal imaging to improve the acquisition speed, making it more suitable for applications that mandate a quicker response, like histological analysis of biopsy samples during surgery. Monitoring the metabolism, differentiation, and disease progress in live cell or tissue cultures could be another valuable venue to explore.

## 4 Appendix

**Fig. 5:**
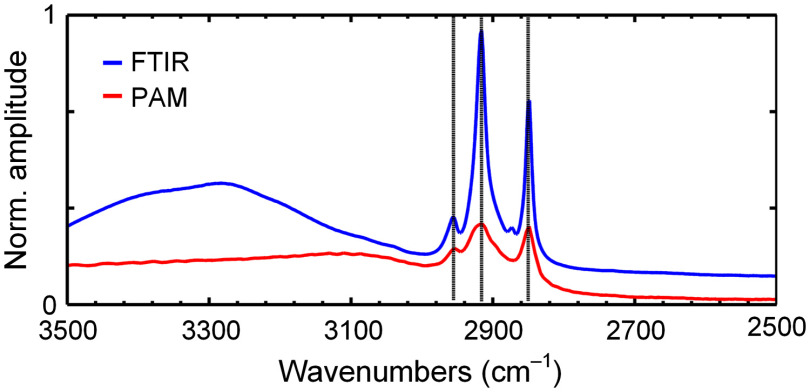
The spectrum of sphingomyelin measured by ATR-FTIR and mid-IR PAM. The vertical lines indicate the main absorption points, showing a good match between the two imaging modalities (difference $<2\;{\mathrm{cm}}^{-1}$). The difference between the two spectra at $∼3300\;{\mathrm{cm}}^{-1}$ may be due to the residual solvent contribution that was not completely subtracted in FTIR.

Figure 5 shows the spectra data of sphingomyelin acquired by a commercial ATR-FTIR spectrometer and by our mid-IR PAM. Good agreement was observed here.

## Code, Data, and Materials Availability

The data that support the plots within this paper and other findings of this study are available from the corresponding author upon reasonable request.
